# University Students’ Perceived Peer Support and Experienced Depressive Symptoms during the COVID-19 Pandemic: The Mediating Role of Emotional Well-Being

**DOI:** 10.3390/ijerph17249308

**Published:** 2020-12-12

**Authors:** Yao Sun, Shiang-Yi Lin, Kevin Kien Hoa Chung

**Affiliations:** 1 Centre for Child and Family Science, The Education University of Hong Kong, Hong Kong, China; sunyaoax@gmail.com (Y.S.); sylin@eduhk.hk (S.-Y.L.); 2 Department of Early Childhood Education, The Education University of Hong Kong, Hong Kong, China

**Keywords:** COVID-19, depressive symptoms, peer support, emotional well-being, loneliness, hope, positive and negative affects

## Abstract

The coronavirus (COVID-19) pandemic has adversely affected individuals’ mental health. Social isolation as a result of social distancing during the pandemic potentially affects the associations among perceived available peer support, emotional well-being, and depression in university students. The present study examined the associations among university students’ perceived available peer support, emotional well-being (as indicated negatively by loneliness and negative affects and positively by positive affects and hope), and depressive symptoms. During the third wave of the COVID-19 outbreak in July, 2020, 255 students at a public university in Hong Kong participated in an online-based survey that assessed their perceived available peer support, emotional well-being, and depressive symptoms. Results showed that perceived available peer support negatively contributed to depressive symptoms; both negative and positive indicators of emotional well-being mediated the association between perceived available peer support and depressive symptoms. Our results also suggested that university students showed signs of elevated depressive symptoms during the pandemic. Thus, our study advanced the theoretical understanding of university students’ mental health in the time of a global pandemic. Our study also highlighted the practical needs for preventive efforts and accessible care to support the psychological and emotional needs of young people during the COVID-19 pandemic.

## 1. Introduction

Since early 2020, the coronavirus disease 2019 (COVID-19) has been a global public health crisis. The disease, first documented in Wuhan, China, quickly spread worldwide and was soon declared a global pandemic by the World Health Organization [1]. While all units in societies have been striving to combat the disease, mental health care for both medical practitioners and the public is desperately needed [2]. Although epidemic control measures such as quarantine and social distancing have helped contain the spread of infections, these measures have also isolated individuals from their social connections and have brought about negative emotions, such as boredom and loneliness [2,3]. To understand the psychological impact of the pandemic, researchers across continents and disciplines gathered data from a wide range of population. Results consistently showed that, since the onset of pandemic, people around the globe have been experiencing a variety of psychological problems, such as depression and anxiety [4,5,6,7]. These problems also emerged among students, given that the majority of schools across the globe were, at least temporarily, shut down, and students were forced to leave schools and stay away from their friends, teachers, and classrooms; their perceptions of peer support—a critical psychological resource—might thus be compromised. Moreover, the isolation and abrupt change of daily routines may lead to poor emotional well-being. Yet, to date, only a handful of studies have attended to university students’ mental health and emotional well-being after school closures [8,9,10,11,12]. Among them, only one Swiss study has investigated university students’ mental health in relation to their perceived available peer support [10].

Thus, to address the paucity of literature on this issue, using data collected from 255 undergraduate students in Hong Kong, the present study aimed to provide insights into university students’ mental health during the COVID-19 pandemic. Specifically, we tested the direct association between students’ perceived available peer support and their depressive symptoms. In addition, we examined the potential mediating roles of four emotional well-being indicators, namely loneliness, negative affects, positive affects, and hope. This attempt is important, as providing adequate psychological support necessitates an in-depth understanding of stressors and mechanisms through which psychological stressors influence mental health.

### 1.1. Depressive Symptoms and Peer Support in the Time of Pandemic

Hong Kong was among the earliest to adopt stringent precautionary measures to contain the COVID-19 pandemic. Nonessential workers have been requested to work from home since late January [13]. Following that, schools were suspended, public places were closed, and citizens were encouraged to stay at home. The city relaxed its social distancing rules after it had kept the disease under control for a while. Schools were briefly reopened in late May, only to be closed several weeks later when the third wave of virus outbreak hit the city (surging new cases had been documented since 22 June). Such prolonged isolation and changes of daily routine are likely to cause psychological and emotional distress among the public and students [4,5,6,7]. One possible reason is that disease control measures such as social isolation make it more difficult for people to satisfy one of their fundamental psychological needs, belongingness [14]. The need of belongingness plays two central roles in maintaining emotional well-being, i.e., the presence of frequent positive contact with others and the perceptions of having stable relationships with others [15]. Unsatisfied need of belongingness leads to negative emotional reactions, which in turn affects mental health [15]. As an immediate result of school closures during the pandemic, students are unable to meet their peers as frequently as before. Social media and other online communications may help increase the sense of social connectedness but are no replacement for face-to-face human contact [16,17]. As students enter adulthood, their primary source of social support is likely to gradually shift from parents to peers [18]. Thus, the abrupt separation from peers during the pandemic may result in social adjustment problems and poor mental health of young adults [12].

Social support helps buffer the negative effects of stressful life events on individuals’ well-being [19]. Social support from peers (for example, having friends to talk to about negative events and feelings), likewise, is an important psychological resource that helps youths combat psychological problems [19,20]. In contrast, the lack of peer support appears to have a negative effect on individuals’ emotional well-being and to exacerbate psychological problems, such as depression. Indeed, a study conducted among African American youths exposed to Hurricane Katrina suggested that perceived greater support from friends was associated with fewer depressive symptoms [20]. One recent study revealed that compared to prepandemic levels, university students nominated fewer fellow students as social interaction partners and perceived less emotional support from their peers during the COVID-19 pandemic; among the same group of students, those who perceived a lower level of emotional support also tended to be more depressed [10].

Guided by both theories of belongingness and social support, this study examined two negative (loneliness and negative affects) and two positive (positive affects and hope) indicators of emotional well-being as potential mediators underlying the relationship between perceived available peer support and depressive symptoms. These particular four emotion-related constructs were selected because they are frequently reported to be emotional factors that contribute to individuals’ psychological problems after exposing to disastrous events, especially the COVID-19 pandemic [21,22,23,24]. Additionally, the emotional indicators of both positive and negative valences were included because extant research has paid little attention to the decrease in positive emotional responses (relative to the rise in negative emotional responses) as a consequence of distress [25]. In the following sections, we reviewed the four constructs and discuss their potential mediating roles in the relationship between perceived available peer support and depressive symptoms.

### 1.2. Loneliness as a Mediator

Loneliness can be seen as an indicator of emotional mal-being due to a state of isolation or lack of companionship [26]. The current social distancing measures, or more accurately, physical separations [14,27], limited individuals’ face-to-face socialization to only family members or coresidents. Enforced isolation may increase individuals’ perceived loneliness and in turn put them at risk for depression. Indeed, as briefly mentioned earlier, research showed that university students experienced a higher level of loneliness during the pandemic (vs. the prepandemic times [10]). In addition to student populations, an increase in loneliness was observed among diverse populations since the onset of pandemic [21,28]). A recent review also concluded that young people were at a high risk of depression due to social isolation and loneliness during the pandemic [29]. Yet, scant study has investigated the role of loneliness in the association between students’ perceived available peer support and their mental health, in the face of a global pandemic. Thus, we suspected that students’ perceived available peer support would be linked to their depressive symptoms during the pandemic via loneliness.

### 1.3. Negative and Positive Affects as Mediators

The prolonged imposed social isolation during the global pandemic catalyzed psychological problems and hurt subjective well-being [30]. Affective responses elicited by the imposed isolation may be one of the underlying mechanisms. Research based on community samples revealed an increase in negative affects (such as anger, boredom, and sadness) and a decrease in positive affects (such as joy, happiness, and satisfaction) during lockdowns or quarantines [5,31]. Recent findings indicated that university students without preexisting mental health problems also showed elevated sadness, reduced joy, and more depressive symptoms during the pandemic, compared to prepandemic levels [9]. In difficult times such as now, having available social support is believed to help assuage unpleasant feelings and prevent individuals from developing psychological problems [19]. Although it appeared that the COVID-19 pandemic had exerted a negative impact on students’ perceived available peer support, affective states, and well-being [23,32], no study has explicitly examined the indirect link between students’ perceived available peer support and depressive symptoms via positive affects or negative affects in the time of pandemic. Thus, both positive and negative affects may potentially mediate the association between students’ perceived available peer support and depressive symptoms during the pandemic.

### 1.4. Hope as a Mediator

Hope is defined as a positive motivational state, fueled by the synergy between two components: a goal-directed energy, or agency, and plans to achieve goals, or pathways [33]. When encountering frustration, hopeful people were motivated to find alternative ways to achieve their goals, whereas less hopeful people tended to ruminate about bad feelings [34]. As a result, being hopeful was associated with better psychological adjustment and lower levels of depression [35]. Peer groups—one of the most relevant social groups to students [18]—seemed to be an essential source of hope. In a meta-analytic study, Mahon and Yarcheski [36] found that peer contact and support were a robust predictor of hope. In line with this view, Stephanou [37] found that preteen students who appraised their peer relationships as more positive, stable, and satisfactory also tended to have more hopeful thinking. In addition, as previous research indicated, when individuals were faced with major adversities in life, such as experiencing an acute trauma (e.g., natural disaster), having solid peer support contributed to individuals’ hopeful thinking and eventually fostered better mental health outcomes [34]. Indeed, it was reported that among a group of Chinese adolescent survivors of the Ya’an earthquake in 2013 [24], perceived social support was associated with hopeful thinking, which in turn led to post-traumatic growth (e.g., better psychological well-being). Although no study has examined the associations among students’ perceived available peer support, hope, and depression during the pandemic yet, we expected a mediating effect to emerge.

### 1.5. The Present Study

To recap, under the impact of COVID-19, individuals around the globe have been experiencing psychological problems such as elevated depressive symptoms [4,5,6,7]. Some public health measures such as school closures and social distancing helped contain the spread of virus, but also prevented people, especially students, from satisfying their need to connect to others. As a result, these measures rendered students isolated and depressed [10]. In addition, available peer support networks during social isolation might help individuals combat psychological problems, such as depression, by improving their emotional well-being [19]. Therefore, several indicators of emotional well-being (such as loneliness) were proposed as potential mediators between perceived available peer support and mental health of student populations.

The present study tested university students’ perceived available peer support, emotional well-being, and mental health during the pandemic. Specifically, we tested the direct association between students’ perceived available peer support and depressive symptoms, as well as the indirect association via loneliness, negative affects, positive affects, and hope, respectively. We hypothesized that university students’ perceived available peer support would be negatively linked to depressive symptoms. We further hypothesized that university students’ perceived available peer support would be negatively associated with loneliness and negative affects, which would contribute to increases in depressive symptoms. Similarly, we hypothesized that university students’ perceived available peer support would be positively associated with hope and positive affects, which would contribute to decreases in depressive symptoms.

## 2. Materials and Methods

### 2.1. Participants and Procedures

Participants of the present study were students from a public university in Hong Kong, China. Through mass emailing, our research team sent out invitation letters to all undergraduate students in the university, introducing our project. We used an online survey platform, Qualtrics, to collect data. The invitation letter students received contained a survey link; by clicking the link, participants were directed to the consent form of our study. Right after students provided written consents, they were then directed to our questionnaire. The purpose of the study was introduced on the first page of the questionnaire, which read “The purpose of the study is to understand university students’ mental health and psychological well-being during the COVID-19, and eventually to provide support to university students’ psychological and emotion needs”. Demographic information was presented first, followed by the measures of depressive symptoms, hope, positive and negative affects, perceived available peer support, and then loneliness. The data were collected from June 6th to July 14th, which overlapped with the onset of the third wave of COVID-19 cases outbreak in Hong Kong [38]. Upon completing the questionnaire, each student received a supermarket coupon of HK$50 (≈US$6) as a token of appreciation. Only the data of those who finished and submitted the online questionnaire were considered as valid responses. Incomplete data (i.e., an omission to submit the questionnaire) were excluded from the analysis. After deleting the data of 51 participants who started the questionnaire but did not submit their responses, the final sample of the study was 255 students. The participated students were asked to provide information on their gender and age. Results showed that our participants’ age ranged from 18 to 35 years old (*M* = 20.96, *SD* = 2.37) and they were predominately female (87%). The procedure of the present study was approved by the institutional review board of The Education University of Hong Kong.

### 2.2. Measures

The original English measures were forward-translated to Chinese and back-translated to English by two bilingual research assistants. A third bilingual researcher checked the translations and resolved the differences.

Perceived available peer support was measured using the 6-item Social Acceptance from Peer subscale of Self-Perception Profile for Children and Adolescents [39]. The scale measured students’ perception of having friends or peers available as a source of social support. In our study, on a 5-point scale (from 1 “strongly disagree” to 5 “strongly agree”), students rated to what extent each statement described their *current* situation. Some example statements were “I have lots of friends,” and “I am always doing things with a lot of peers”. Total scores were calculated by averaging the scores of all items. The scores on average were 3.24 (*SD* = 0.43; Table 1). A higher score indicated a higher level of perceptions of available peer support. The Cronbach’s alpha for the scale was 0.66.

Depressive symptoms were measured using the 10-item Center for Epidemiologic Studies Depression scale (CES-D-10 [40]). This scale was commonly used in adolescents and young adults to measure their experience of depressive symptoms. The CES-D-10 tapped into a range of depressive symptoms, such as depressed mood, sleep disturbance, and feelings of worthlessness. In our study, on a 4-point scale, ranging from 0 (rarely or none of the time, less than 1 day per week) to 3 (all of the time, 5–7 days per week), students indicated the frequency of experiencing depressive symptoms described in each item. A prompt was added to the scale instruction that directed participants to answer each item considering their well-being during the COVID-19 pandemic. Some example items were “I had trouble keeping my mind on what I was doing” and “I felt depressed”. Total scores were calculated by averaging the scores of all items. The scores on average were 1.13 (*SD* = 0.60; Table 1). A higher score indicated a greater level of depressive symptoms. The Cronbach’s alpha was 0.89.

Positive and negative affects were measured using the 10-item Positive and Negative Affect Scale—Short Form (PANAS [41]). This scale contained 5 positive emotion words and 5 negative emotion words. In our study, on a 5-point scale, ranging from 1 (very slightly or not at all) to 5 (a lot or often), students were asked to rate the extent to which each emotion word described their affective state at the present moment. Example items for positive affects were “inspired” and “active”, and example items for negative affects were “upset” and “afraid”. The mean scores of positive affects and negative affects were calculated separately. The scores on average for positive and negative affects were 2.89 (*SD* = 0.65) and 2.56 (*SD* = 0.86; Table 1). A higher score in positive affects or negative affects indicated a higher level of the respective constructs. The Cronbach’s alpha for positive affects and negative affects were 0.78 and 0.87, respectively.

Loneliness was measured using the 6-item Brief Scale of Loneliness [42]. The brief scale was a self-report measure that emphasizes feelings of loneliness in a social context. Students rated their *current* perceived loneliness on a 5-point scale, ranging from 1 (never) to 5 (often). Some example items were “I feel left out,” and “people are around me but not with me”. Total scores were calculated by averaging the scores of all items. The scores on average were 2.67 (*SD* = 0.76; Table 1). A higher score indicated a higher level of loneliness. The Cronbach’s alpha was 0.86.

Hope was measured using 6-item State Hope Scale [43]. The State Hope Scale, distinct from the Trait Hope Scale, measures individuals’ hopeful thinking right now [34]. Because the aim of our study was to capture students’ well-being at the present moment, the State Hope Scale was adopted. On a 4-point scale, from 1 (definitely false) to 5 (definitely true), students rated to what extent each statement was descriptive of themselves. Some example statements were “If I should find myself in a jam, I could think of many ways to get out of it,” and “There are lots of ways around any problem that I am facing now”. Total scores were calculated by averaging the scores of all items. The scores on average were 2.76 (*SD* = 0.47; Table 1). A higher score represented a higher level of hopeful thinking. The Cronbach’s alpha was 0.86.

## 3. Results

Data were analyzed using SPSS statistics (version 26, IBM, Armonk, NY, USA). The results of descriptive statistics and Pearson’s correlations (two-tailed) were summarized in Table 1. Notably, the mean score of students’ self-rated depressive symptoms (*M* = 1.13) was higher than the consensus cutoff score for clinically significant depressive symptoms (cutoff mean score = 1 [44,45]). We further probed into the distribution of the mean scores; the results of frequency analysis showed that more than half (56.9%) of the participants scored higher than the cutoff score, suggesting that students experienced significant depressive symptoms during the pandemic. Returning to the correlations with depressive symptoms, as hypothesized, results showed that perceptions of available peer support were negatively correlated with depressive symptoms (*r* = −0.32, *p* < 0.01). In other words, as predicted, students who perceived greater peer support had fewer depressive symptoms. All four indicators of emotional well-being—loneliness (*r* = 0.45, *p* < 0.01), negative affects (*r* = 0.64, *p* < 0.01), positive affects (*r* = −0.27, *p* < 0.01), and hope (*r* = −0.51, *p* < 0.01) were also associated with depressive symptoms. Interestingly, positive affects were not related to negative affects (*r* = −0.04, *p* = 0.517), suggesting that they are distinct constructs instead of opposing qualities of one construct [46]. 

Next, using Hayes’s [47] PROCESS macro (model 4) in SPSS, we tested loneliness, negative affects, positive affects, and hope as the mediators between students’ perceived available peer support and depressive symptoms. To this end, we first entered students’ age and gender as covariates, as these demographic characteristics were previously linked to university students’ depression [48]. Gender was dummy-coded: 0 represented female and 1 represented male. Then, perceived available peer support was entered as the independent variable, and depressive symptoms was entered as the dependent variable. Finally, loneliness, negative affects, positive affects, and hope were entered simultaneously as parallel mediators. Following the recommendation of Preacher and Hayes [49], 10,000 bootstrapping samples and 95% confidence intervals (CIs) were adopted to test mediation effects and evaluate their statistical significance [50]. A mediation effect was considered significant if the 95% CI excluded zero.

The overall mediation model—with the independent variable and all the mediators as predictors—explained 54% of the total variance of the dependent variable. As shown in Table 2, bootstrapping results of the overall mediation model (*β* = −0.28, *SE* = 0.06, 95 % CI (−0.39, −0.17)) excluded zero. Moreover, bootstrapping results of the four mediators—loneliness (*β* = −0.09, *SE* = 0.04, 95 % CI (−0.17, −0.02)), negative affects (*β* = −0.09, *SE* = 0.03, 95 % CI (−0.15, −0.02)), positive affects (*β* = −0.05, *SE* = 0.02, 95 % CI (−0.10, −0.00)), and hope (*β* = −0.06, *SE* = 0.03, 95 % CI (−0.11, −0.01)) all excluded zero.

**Figure 1:**
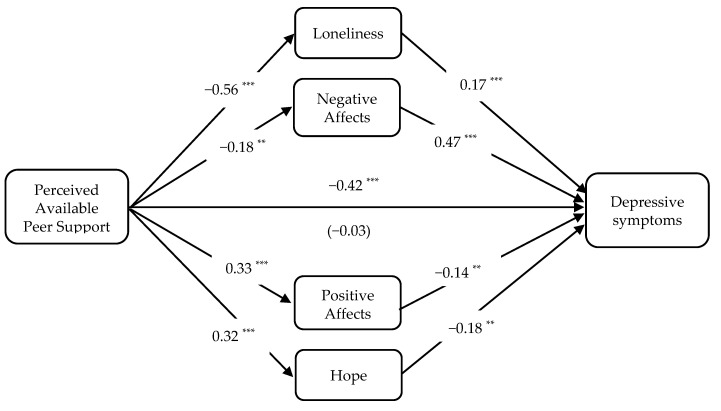
The direct effect (standardized coefficients) of each path. Note: ** *p* < 0.01, and *** *p* < 0.001. Direct effect of perceived available peer support on depressive symptoms after introduced mediators was presented in parenthesis. Gender and age were entered as covariates (not presented in the figure).

Effects by paths were also examined and presented in Figure 1. After introducing the mediators into the model, the direct association between students’ perceived available peer support and depressive symptoms were nonsignificant (*β* = −0.03, *SE* = 0.08, *p* = 0.687). Apart from this, all paths presented were significant and in the expected directions. Specifically, perceived available peer support was negatively associated with loneliness (*β* = −0.56, *SE* = 0.09, *p* < 0.001) and negative affects (*β* = −0.18, *SE* = 0.12, *p* = 0.003), which further linked to more depressive symptoms (loneliness: *β* = 0.17, *SE* = 0.05, *p* = 0.004; negative affects: *β* = 0.47, *SE* = 0.04, *p* < 0.001). Similarly, perceived available peer support was positively linked to positive affects (*β* = 0.33, *SE* = 0.09, *p* < 0.001) and hope (*β* = 0.32, *SE* = 0.06, *p* < 0.001), which then were associated with fewer depressive symptoms (positive affects: *β* = −0.14, *SE* = 0.05, *p* = 0.008; hope: *β* = −0.18, *SE* = 0.07, *p* = 0.002).

## 4. Discussion

There has been a growing concern for the impacts of the pandemic on people’s mental health. Indeed, fear for the spread of the deadly virus and sense of isolation due to social distancing have marked this difficult time. To address this concern, several studies have reported to investigated, and proposed means to ameliorate, individuals’ mental health during the pandemic [4,5,6,7]. Although these investigations have provided timely insights, most of them did not examine the associations between relational factors, such as perceived available peer support, and psychological problems, such as depression. Thus, the first aim of our study was to test the direct link between university students’ perceived available peer support and depressive symptoms they experienced during the pandemic. Moreover, although the public has reported a series of emotional distress [21,22,23], the role of individuals’ emotional well-being in relation to their perceived relational support and mental health has rarely been examined. Hence, the second aim of our study was to test the mediating effects of the indicators of emotional well-being (i.e., loneliness, negative and positive affects, and hope).

Aligned with our first hypothesis, the results yielded a negative association between students’ perceived available peer support and their self-reported depressive symptoms. This result was in line with the notion that prolonged social isolation measures during COVID-19 pandemic may have prevented people from satisfying one of their basic needs—the needs to belong and to connect with others [14,15]. Given that many schools remained closed for months, students may have had fewer chances to interact with their friends at schools and consequently felt less connected with their peers [10]. Our findings underscored the important role of relational factors, such as perceived available peer support, for students’ mental health in the time of the pandemic. Education stakeholders should help enhance students’ perception of social support from and connectedness with peers without violating current disease control measures, for example, by encouraging students to communicate with peers and close others virtually using social media [12,16].

Nevertheless, the association between students’ perceived available peer support and their self-reported depressive symptoms was robustly mediated by the four indicators related to emotional well-being (i.e., loneliness, negative affects, positive affects, and hope). Indeed, as suggested by social support theory, support network helped individuals combat psychological problems due to disasters through promoting their emotional well-being [19,20]. Thus, our second and third hypotheses were also supported. Regarding the negative indicators of emotional well-being, higher levels of perceived available peer support were associated with less loneliness and fewer negative affects, which were further related to greater depressive symptoms. Such results were in accordance with recent findings showing that university students felt less supported by their peers, lonelier, sadder, and had more psychological problems during the pandemic (vs. before the pandemic [9,10]). With regard to the positive indicators of emotional well-being, similarly, high levels of perceived available peer support were associated with higher levels of positive affects and hope, both of which were associated with fewer depressive symptoms in university students. Again, these findings were in line with the conclusions from previous studies that positive affects and hopeful thinking were derived from positive interpersonal relationships and contributed to better psychological adjustment of individuals [35,37]. Notably, although hope was documented as an important mediator of earthquake survivors’ social support and psychological adjustment [24], hopelessness [51,52], but not hope, was more frequently studied during the pandemic. Thus, another contribution of our study was linking university students’ emotional well-being, especially senses of hope, to their perceived available peer support and mental health during the pandemic.

Taken together, our findings lend support to the notion that high levels of perceived available support from peers help buffer psychological problems during difficult times, by improving the emotional well-being of individuals [19,20]. In particular, our finding pointed to the fact that both positive and negative affects were influenced as a result of prolonged social isolation. Albeit the proposition that positive emotions should be considered as a unique dimension of emotion—rather than the opposing quality of negative emotions [46]—(a decrease of) positive emotions was not widely studied as a consequence of distress [25]. Thus, another strength of the present study was the inclusion of both negative emotion-related constructs (negative affects and loneliness) and positive emotion-related constructs (positive affects and hope) as indicators of emotional well-being. In this regard, a potential future direction is to further expand the scope of the positive and negative emotion-related constructs, for example, including several emotion-related constructs that were frequently reported by the public during the pandemic, e.g., fear [53,54], boredom [55,56], and nostalgia [57,58].

Our study also bears important practical implications. The present study shed light on university students’ mental health during the pandemic. Specifically, our findings highlighted the immediate relevance of students’ perceptions of available peer support to their mental health and the mediating effect of their emotional well-being. In addition, the majority of our sample scored high on the depressive symptoms scale, which seemed to have corroborated concerns about the poor mental health condition of the public, especially young people, during the pandemic [2,3,59]. Indeed, although the stringent disease control measures—social distancing, school closures, and shutdown of public facilities—have helped contained the virus, they also took a toll on young people’s mental health.

Even though young people may not be contributing to the COVID-19 death toll, they are hugely impacted otherwise [12,29]. According to a national survey of Center for Disease Control and Prevention (CDC), more than 40% of US young adults reported to have experienced symptoms of depression or anxiety during the pandemic [60]. The unemployment rate has risen, exchange programs were cancelled, and scholarship opportunities were slashed, all of which are likely to worsen young people’s mental well-being [61]. Thus, more studies that focus on university students’ relational, emotional, and psychological health are needed [9,11]. Likewise, preventive intervention programs or other—preferably online—resources should be made available and accessible to students, in order to strengthen their emotional well-being and to reduce their psychological distress due to the COVID-19 [62].

Our study is not without limitation, however. To begin with, our data were collected from a group of students at only one university in Hong Kong, which has a disproportionate female population. In other words, our sample cannot represent all university students in Hong Kong, not to mention individuals of other age groups or from other countries. Thus, generalization of our findings should be made with caution. Secondly, students themselves were the sole informants in the present study, which may potentially introduce a method bias [63]. Our findings need to be replicated using multiple methods and to collect data from multiple informants. Thirdly, our cross-sectional design and correlational analysis prevented us from making causal inferences. Additionally, due to our lack of prepandemic data, we are unable to rule out the possibility that our findings can be explained by students’ earlier levels of depression (e.g., students might have already been depressed before the pandemic). To address these issues, future researchers should adopt a more vigorous design, such as collecting data across multiple time points, and comparing pre- and post-pandemic mental health data [64]. Fourthly, although we specified in the instructions that the participants should answer questions considering their current condition or their condition since the onset of the pandemic, we did not directly measure their perceptions of the pandemic. Thus, future studies would benefit by including questions that explicitly ask students about their perceptions of the pandemic and by trying to link their perceptions of the pandemic with their mental health, such as how much their lives have been (negatively) impacted by the pandemic or how worried they are about the pandemic. Finally, in the present study, we measured students’ perceptions of their available peer support network in terms of having friends available to provide peer support (“I have lots of friends”) and being surrounded by peers (“I am always doing things with a lot of peers”). Despite its relevance to the present study, this measure did not cover specific kinds of support (e.g., emotional support, instrumental support), nor did it touch on other sources of social support (e.g., parents, teachers). Thus, future studies need to include measures that cover broader aspects of social support.

## 5. Conclusions

In light of these strengths and limitations, the present study suggested that university students’ perceived available peer support was negatively associated with their depressive symptoms during the pandemic. Further, our study identified four emotional wellbeing indicators that underlie this association, namely loneliness, positive and negative affects, and hope. Our findings have a number of important implications for future research. In particular, the findings advance the understanding of the mental health and emotional well-being of university students during the pandemic. The findings also provide valuable information for potential psychological consequences following governments’ preventive measures to combat the disease.

## Figures and Tables

**Table 1:** Descriptive statistics and correlations of variables.

Variables	1	2	3	4	5	6	7	*M*	*SD*	Range
1. Age	-	0.25 **	−0.06	0.11	0.18 **	−0.04	−0.11	20.96	2.37	18–35
2. Depressive symptoms		-	−0.32 **	0.45 **	0.64 **	−0.27 **	−0.51 **	1.13	0.60	0–3
3. Perceived Available Peer Support			-	−0.57 **	−0.20 **	0.33 **	0.32 **	3.24	0.43	1–5
4. Loneliness				-	0.41 **	−0.11	−0.31 **	2.67	0.76	1–5
5. Negative Affects					-	−0.04	−0.42 **	2.56	0.86	1–5
6. Positive Affects						-	0.45 **	2.89	0.65	1–5
7. Hope							-	2.76	0.47	1–4

Note: ** *p* < 0.01.

**Table 2:** Standardized coefficients and bootstrapping results of the mediation model.

Mediator	*β*	*SE*	95 % Confident Interval
Total	−0.28	0.06	(−0.39, −0.17)
Loneliness	−0.09	0.04	(−0.17, −0.02)
Negative Affects	−0.09	0.03	(−0.15, −0.02)
Positive Affects	−0.05	0.02	(−0.10, −0.00)
Hope	−0.06	0.03	(−0.11, −0.01)

Note: For each path, the independent variable was perceived available peer support and the dependent variable was depressive symptoms. Gender and age were controlled for as covariates. A mediation effect was considered significant if the 95 % CI excluded zero.
